# Leveraging artificial intelligence to explore gendered patterns in financial literacy among teachers in academia

**DOI:** 10.3389/frai.2025.1634640

**Published:** 2025-09-24

**Authors:** A. Ruban Christopher, A. R. Nithya

**Affiliations:** School of Management, Hindustan Institute of Technology and Science, Chennai, India

**Keywords:** artificial intelligence, financial literacy, gender disparities, academia, machine learning, natural language processing, socioeconomic context

## Abstract

**Introduction:**

Financial literacy is essential for long-term economic stability, yet persistent gender disparities in financial knowledge continue to be observed across professions, including academia. This study explores how Artificial Intelligence (AI) can be applied to identify and analyze gender-based patterns in financial literacy among higher education faculty.

**Methods:**

A mixed-methods design was employed, combining traditional survey instruments with AI-driven analytics. Survey data were collected from 300 academic professionals across diverse institutions, capturing financial knowledge, attitudes, behaviors, and socioeconomic characteristics such as marital status, number of dependents, and family income. Natural language processing (NLP) and machine learning (ML) techniques were used to detect linguistic and behavioral differences between male and female participants.

**Results:**

Findings revealed statistically significant gender gaps in financial literacy. Male participants scored higher in investing knowledge (Δ=1.9 points, p<0.001) and expressed greater confidence (+0.42 sentiment vs. -0.15 for women). Intersectional analysis showed that women in STEM disciplines demonstrated narrower gaps (Δ=0.7) compared to women in the humanities (Δ=1.2), with disparities shaped by wage differentials and caregiving responsibilities. Socioeconomic factors—including marital status, family size, and income—were also associated with variations in financial literacy and investment confidence. While the findings are correlational, AI-powered sentiment and cluster analyses provided deeper insights into behavioral segments, illustrating the compounded influence of gender, discipline, and socioeconomic context.

**Discussion:**

By integrating AI techniques with traditional survey methods, this research advances the study of gender and financial literacy in academia. The combined approach enhances interpretability and highlights the value of context-sensitive interventions. Recommended strategies include gender-responsive financial training, AI-enabled coaching tools, and institutional and policy-level reforms supported by universities, government agencies, and funding bodies.

## 1 Introduction

Financial literacy—defined as the ability to understand and apply financial concepts such as budgeting, investing, and debt management—plays a crucial role in personal and professional economic stability (Lusardi and Mitchell, 2014). Despite its importance, research consistently highlights gender disparities in financial literacy, with women often scoring lower than men in assessments of both knowledge and confidence (Bucher-Koenen et al., 2017). These disparities persist even among highly educated professionals, including teachers in academia, where financial decision-making impacts career longevity, retirement planning, and economic resilience (Gerrans et al., 2014).

The integration of Artificial Intelligence (AI) in social science research presents a significant opportunity to explore these disparities with greater analytical precision. Traditional surveys and interviews, while valuable, may overlook subtle linguistic and behavioral differences that AI-driven techniques—such as Natural Language Processing (NLP), sentiment analysis, and machine learning (ML) clustering—can detect linguistic and behavioral differences (Argyle et al., 2023; Bolukbasi et al., 2016; Bird et al., 2009; Grimmer and Stewart, 2013; Kozlowski et al., 2019; Hutto and Gilbert, 2014; Li et al., 2022). By leveraging AI, this study uncovers hidden gendered patterns in financial literacy that conventional methods might miss, particularly in relation to the socioeconomic context of the respondents, including marital status, number of children, and family income level.

In an era marked by rapid advancements and societal shifts, academia is not merely a harbinger of knowledge but also a mirror reflecting societal dynamics. Among these, gender equity and financial literacy emerge as critical pillars shaping the educational landscape. Gender equity, rooted in the ethos of fair and just treatment for all genders, has evolved to transcend the simplistic notion of equality, aiming instead to address varied needs, lived experiences, and historical contexts (International Labour Organization, 2020). In academia, gender equity initiatives aim to create an environment in which educators, irrespective of gender, can thrive and contribute equitably.

Financial literacy, in this context, encompasses more than personal money management; it extends to understanding institutional finance, retirement systems, and long-term wealth planning. For educators, particularly in resource-constrained settings, financial literacy influences not only their own economic well-being but also their ability to guide students in making informed financial decisions Organization for Economic Co-operation and Development (OECD, 2020). Previous research has demonstrated a strong correlation—not causation—between financial literacy and gender equity, suggesting that improving financial knowledge can help reduce gender-based disparities in professional and personal outcomes (Bucher-Koenen et al., 2017; Kaiser and Menkhoff, 2017).

However, financial literacy gaps are not driven by gender alone. Studies have shown that marital status, caregiving responsibilities, and household income levels intersect with gender to influence financial behaviors (Fonseca et al., 2012; Hsu, 2016). Married women with children, for example, often exhibit more risk-averse investment strategies, not solely due to personal preference but also because of household financial priorities and societal expectations (Agnew et al., 2013). This highlights the importance of including such socioeconomic variables in analyses, as they provide a richer, more accurate understanding of observed patterns.

The city of Chennai, with its blend of traditional socio-cultural norms and emerging economic opportunities, offers a compelling context for studying these dynamics. In this environment, female educators frequently navigate dual pressures: the demands of professional advancement and the expectations of familial financial contribution. These intersecting factors make the exploration of gendered financial literacy especially relevant, as the cultural framework may moderate or amplify existing disparities (Sharma and Zeller, 2023).

This paper therefore addresses a crucial research gap by combining traditional survey-based methods with AI-enhanced analytics to investigate financial literacy in academia. AI techniques enable deeper interrogation of qualitative data, identifying sentiment and lexical patterns that correlate with financial literacy scores, while statistical models incorporate demographic, professional, and socioeconomic factors. This mixed-method approach enriches our understanding of how gendered patterns manifest in both linguistic and behavioral domains.

The specific objectives of the study are:

1. To assess gender-based differences in financial literacy among academic professionals in Chennai, considering both professional characteristics and socioeconomic factors such as marital status, number of children, and family income.
2. To apply AI-driven NLP and sentiment analysis to qualitative responses, identifying latent behavioral and attitudinal cues.
3. To explore intersectional influences of discipline, academic rank, and household characteristics on financial knowledge and behavior.
4. To propose targeted, actionable interventions for improving financial literacy, grounded in both statistical evidence and AI-derived insights.

The findings are correlational in nature, acknowledging the limitations of cross-sectional data and the inability to infer cause-and-effect relationships. Nonetheless, by integrating AI analytics with established survey methodologies, this study offers both methodological innovation and practical implications. The outcomes contribute to the discourse on gender equity, financial inclusion, and the role of AI in enhancing social science research.

## 2 Literature review

### 2.1 Financial literacy: definitions and importance

Financial literacy is widely defined as the knowledge and skills necessary to make informed and effective decisions regarding financial resources, encompassing budgeting, savings, investment, credit management, and retirement planning (OECD, 2020). Higher financial literacy has been consistently associated with improved long-term decision-making and greater economic resilience (Klapper and Lusardi, 2020).

However, empirical research across multiple contexts reveals persistent gender gaps in financial literacy. Studies consistently highlight persistent gender gaps in financial literacy across contexts (Arrondel et al., 2013; Barboza et al., 2014; Brown and Graf, 2013; Hastings et al., 2013; Lührmann et al., 2015; Rooij et al., 2011). Women, on average, tend to score lower on standardized financial literacy measures than men (Lusardi and Mitchell, 2014; Potrich et al., 2018). These differences have been linked to multiple factors, including risk aversion (Sundén and Surette, 1998), lower self-confidence in financial decision-making (Bucher-Koenen et al., 2017), and socio-cultural norms shaping financial roles (Fonseca et al., 2012).

Importantly, financial literacy is also shaped by socioeconomic variables—including marital status, number of dependents, and household income—which interact with gender to produce distinct financial behaviors (Hsu, 2016; Agnew et al., 2013). Married women, particularly those with children, often prioritize financial security and short-term liquidity over high-return investment options, reflecting both household needs and cultural expectations (Sharma and Zeller, 2023).

### 2.2 AI in financial literacy research

Advances in AI have introduced new opportunities for studying financial literacy patterns beyond traditional statistical approaches. NLP and ML techniques can detect latent linguistic patterns, sentiment orientations, and behavioral clustering that are difficult to capture through structured survey items (Hutto and Gilbert, 2014; Li et al., 2022).

For instance, AI-driven sentiment analysis has been employed to evaluate confidence and risk perceptions in financial discourse, identifying gendered expressions such as “confident” versus “unsure” (Caliskan et al., 2017). Similarly, clustering algorithms can segment populations into behavior-based groups—such as high-risk investors or conservative savers—based on multidimensional input data (Bishop, 2006; Mehrabi et al., 2021).

Despite these advancements, the integration of AI into gender-focused financial literacy research remains limited. Few studies apply AI methods alongside intersectional demographic analysis, leaving an important gap that this study addresses.

### 2.3 Gender, socioeconomic context, and financial behavior

Gendered financial behaviors cannot be explained by gender identity alone. Intersectionality theory (Crenshaw, 1989) posits that overlapping social categories—such as gender, marital status, parental responsibilities, and economic class—interact to create unique experiences and disparities. Within academia, these intersections are evident in pay differentials between disciplines, variable access to institutional resources, and differing caregiving responsibilities, all of which correlate with financial confidence and decision-making (McCall, 2005; Roy and Patro, 2022).

Research on financial inclusion emphasizes the barriers faced by women in accessing financial education and resources, with particular challenges for those balancing professional roles with household responsibilities (Fonseca et al., 2012; Andarsari and Ningtyas, 2019). Income level has been shown to moderate these effects: higher-income households tend to engage more in long-term financial planning, while lower-income households focus on immediate needs (OECD, 2020).

Studies in the Indian context reveal additional layers, where traditional gender norms influence the allocation of financial responsibilities within households (Goyal and Kumar, 2021). For female academics, these dynamics often intersect with institutional hierarchies, creating compounded disadvantages.

### 2.4 Empirical studies in academic contexts

Several studies have examined financial literacy among educators and students, offering insights into the gender gap:

- López Hernández (2022) found that targeted financial education programs in academic institutions significantly improved decision-making confidence among female faculty members.
- Mändmaa (2020) reported persistent gender gaps among university students, despite equal academic performance, suggesting that socialization patterns rather than intellectual capacity drive disparities.
- Sahabuddin and Hadianto (2023) observed that female students exhibited more conservative investment behaviors, correlating with self-reported lower confidence and higher risk aversion.
- Getmansky Sherman and Tookes (2021) highlighted the underrepresentation of women in finance academia, linking this to gaps in mentorship and institutional support.

Early interventions are particularly critical. Migheli and Coda Moscarola (2017) demonstrated that financial education introduced at the primary school level can substantially reduce gender gaps in later years. Similar evidence from Kaiser and Menkhoff (2017) suggests that well-designed financial education programs have the strongest impact when delivered early and tailored to specific needs.

### 2.5 Research gap

While a growing body of literature examines gender disparities in financial literacy, significant gaps remain:

1. Limited use of AI for analyzing qualitative aspects of financial literacy, such as sentiment, lexical choice, and behavioral clustering.
2. Insufficient integration of socioeconomic context variables—marital status, number of children, and family income—into analytical models, despite evidence of their influence on financial behavior.
3. Lack of intersectional analysis that combines gender with academic rank, discipline, and household characteristics in a single framework.

This study addresses these gaps by employing a mixed-method, AI-augmented approach to examine financial literacy among academics in Chennai. It incorporates comprehensive demographic and socioeconomic data, applies NLP and sentiment analysis to qualitative responses, and uses clustering algorithms to identify behavioral profiles. The findings are interpreted within a correlation-based framework, offering nuanced insights into how gender and socioeconomic contexts interact to shape financial literacy in academia.

## 3 Research methodology

This study adopted a structured, mixed-method research design to examine gendered patterns in financial literacy among teachers in Chennai, Tamil Nadu, India, integrating both quantitative and qualitative approaches. The design leveraged AI techniques to complement traditional statistical analysis, enabling richer interpretation of linguistic and behavioral differences between male and female participants.

### 3.1 Sample size and selection

A total of 300 academic professionals participated in the study, representing a diverse cross-section of educational institutions, disciplines, and career stages. The sample was stratified to ensure representation across gender, academic rank, and institutional type (public, private, autonomous). In addition to gender, participants were asked to report their marital status (single, married, divorced/widowed), number of children/dependents, and monthly household income. These socioeconomic variables were included to provide deeper insight into the contextual factors influencing financial literacy.

Sampling technique:

- Stratified random sampling ensured balanced representation of male and female educators across disciplines (Science, Technology, Engineering, and Mathematics [STEM] and Humanities) and ranks (assistant, associate, and full professors).
- Socioeconomic strata were incorporated to account for diversity in family responsibilities and income levels.

Geographical area: The study focused on the urban and suburban areas of Chennai, where academic institutions range from large universities to smaller private colleges, offering a blend of contexts for analysis.

### 3.2 Data collection

Data were collected between September 2024 and November 2024 using a combination of online surveys (via secure forms) and in-person questionnaires. This dual-mode approach increased accessibility and participation rates, particularly among senior faculty less inclined toward online platforms. The overall response rate was 80%, indicating strong engagement. Data Collection Team: A team of trained researchers proficient in both English and Tamil facilitated the process, ensuring clarity of communication and cultural appropriateness in interactions.

### 3.3 Research instrument

The survey instrument was adapted from the OECD (2020) financial literacy framework, contextualized for academic professionals in India. The instrument contained four sections:

1. Demographics and socioeconomic data: Gender, age, marital status, number of children, household income, educational qualification, academic discipline, and rank.
2. Financial knowledge assessment: A 10-point scale measuring understanding of budgeting, investing, risk management, and retirement planning (e.g., “How confident are you in your ability to create a long-term investment plan?” rated from 1 = Not confident to 10 = Very confident).
3. Financial behavior measures: Frequency of reviewing retirement savings, investment diversification, sources of financial advice, and self-reported risk tolerance.
4. Open-ended qualitative questions: Eliciting descriptions of recent financial decisions, influencing factors, and perceived challenges (e.g., “Describe a recent financial decision you made and the factors that influenced it.”).

The inclusion of marital status, number of dependents, and household income allowed for an intersectional analysis of financial literacy patterns. For instance, we examined whether married women with dependents exhibited different investment behaviors than single women without dependents, and how these differences correlated with income levels.

### 3.4 Data preparation and reliability

- Missing data handling: Listwise deletion was used for incomplete quantitative responses to preserve statistical validity. Median imputation addressed isolated missing values in Likert-scale items.
- Qualitative data screening: Short or unclear open-ended responses were excluded from AI-driven text analysis to avoid distortion in sentiment and keyword trends.
- Reliability: Internal consistency of the financial literacy scale was confirmed with Cronbach’s alpha = 0.82.
- Validity: Expert review ensured both content and construct validity, aligning items with the study objectives and local context.

### 3.5 Statistical analysis

- Descriptive statistics: Used to summarize demographic and socioeconomic variables, as well as financial literacy scores.
- Inferential statistics: Independent samples *t*-tests and one-way ANOVA examined group differences by gender, discipline, rank, marital status, number of dependents, and income level. Effect sizes (Cohen’s *d*) were calculated to interpret the magnitude of observed differences.
- Correlation analysis: Pearson’s correlation coefficient (*r*) assessed associations between financial literacy and continuous variables such as household income and number of dependents. All findings were interpreted as associations rather than causal relationships due to the cross-sectional nature of the data.

### 3.6 AI-driven analytical methods

Qualitative responses were processed using Python’s Natural Language Toolkit (NLTK) and the VADER sentiment analysis tool.

Pre-processing steps included:

- Lowercasing and punctuation removal.
- Tokenization and stop-word filtering.
- Lemmatization using WordNetLemmatizer.
- Manual review to remove irrelevant or ambiguous phrases.

#### 3.6.1 Sentiment analysis

VADER was selected for its high interpretability in short-text sentiment scoring, particularly suited for mixed formal-informal language common in academic discourse. Sentiment polarity scores ranged from −1 (strongly negative) to +1 (strongly positive).

#### 3.6.2 Keyword frequency analysis

Weighted term frequencies were calculated by gender and socioeconomic group allowing comparisons such as “confident” (more common among high-income men) versus “safe” (more common among married women with children).

#### 3.6.3 Cluster analysis

- Conducted with K-means clustering on standardized variables: financial literacy score, investment confidence, and risk tolerance.
- Optimal cluster number (*k* = 3) determined using the elbow method and silhouette score (0.62).
- Each cluster was profiled by gender, marital status, number of dependents, income level, discipline, and academic rank.
- SHAP (SHapley Additive exPlanations) values identified the top predictors of cluster membership (e.g., institutional financial training, income, caregiving responsibilities).

### 3.7 Ethical considerations

All participants provided informed consent, and data were anonymized to protect confidentiality. The study complied with the principles outlined in the Declaration of Helsinki.

## 4 Findings and analysis

This section presents the statistical and AI-driven findings from the survey, integrating both quantitative and qualitative results. All relationships discussed are correlational and interpreted with caution due to the cross-sectional nature of the data.

### 4.1 Socio-demographic characteristics of respondents

Caption: Respondent demographics, including socioeconomic factors (marital status, number of children, and income), highlighting a diverse mix of participants across age groups, education levels, and household structures (Table 1).

**Table 1:** Socio-demographic characteristics of the respondents.

Demographic feature	Frequency count	Percentage (%)
Gender: Male	150	50.0%
Gender: Female	150	50.0%
Age: Under 25	45	15.0%
Age: 25–34	105	35.0%
Age: 35–44	85	28.3%
Age: 45–54	40	13.3%
Age: 55 or older	25	8.3%
Bachelor’s degree	90	30.0%
Master’s degree	150	50.0%
Ph. D. or Advanced Degree	60	20.0%
Married	198	66.0%
Single	88	29.3%
Divorced/Widowed	14	4.7%
No children	122	40.7%
One child	96	32.0%
Two or more children	82	27.3%
Monthly Household Income < ₹50,000	86	28.7%
₹50,000 – ₹1,00,000	138	46.0%
Above ₹1,00,000	76	25.3%

Interpretation: A balanced gender split was achieved, ensuring comparability between male and female participants. Most respondents were married (66%), and nearly 60% had at least one child—variables that could influence financial decision-making priorities. Household income distribution suggests that nearly half of the sample fell into the middle-income bracket (₹50,000–₹1,00,000 per month), which may shape investment capabilities and risk tolerance.

### 4.2 Overall financial literacy scores

Caption: Mean financial literacy scores by gender, based on OECD (2020) framework, showing statistically significant differences (Table 2).

**Table 2:** Financial literacy scores by gender.

Gender	Mean score (SD)	*t*-value	*p*-value
Male	7.2 (1.4)	4.83	<0.001
Female	6.1 (1.6)		

Interpretation: Men scored higher on average than women, with a difference of 1.1 points (*p* < 0.001). This gender gap persisted after controlling for marital status, number of children, and income, though the magnitude was smaller among high-income households. Married women with two or more children scored, on average, 0.6 points lower than unmarried women without children—indicating a potential link between caregiving responsibilities and lower financial literacy scores.

### 4.3 Subdomain analysis

Financial literacy was further broken down into:

1. Budgeting and saving.
2. Investing and risk management.
3. Retirement planning.

**Figure 1:**
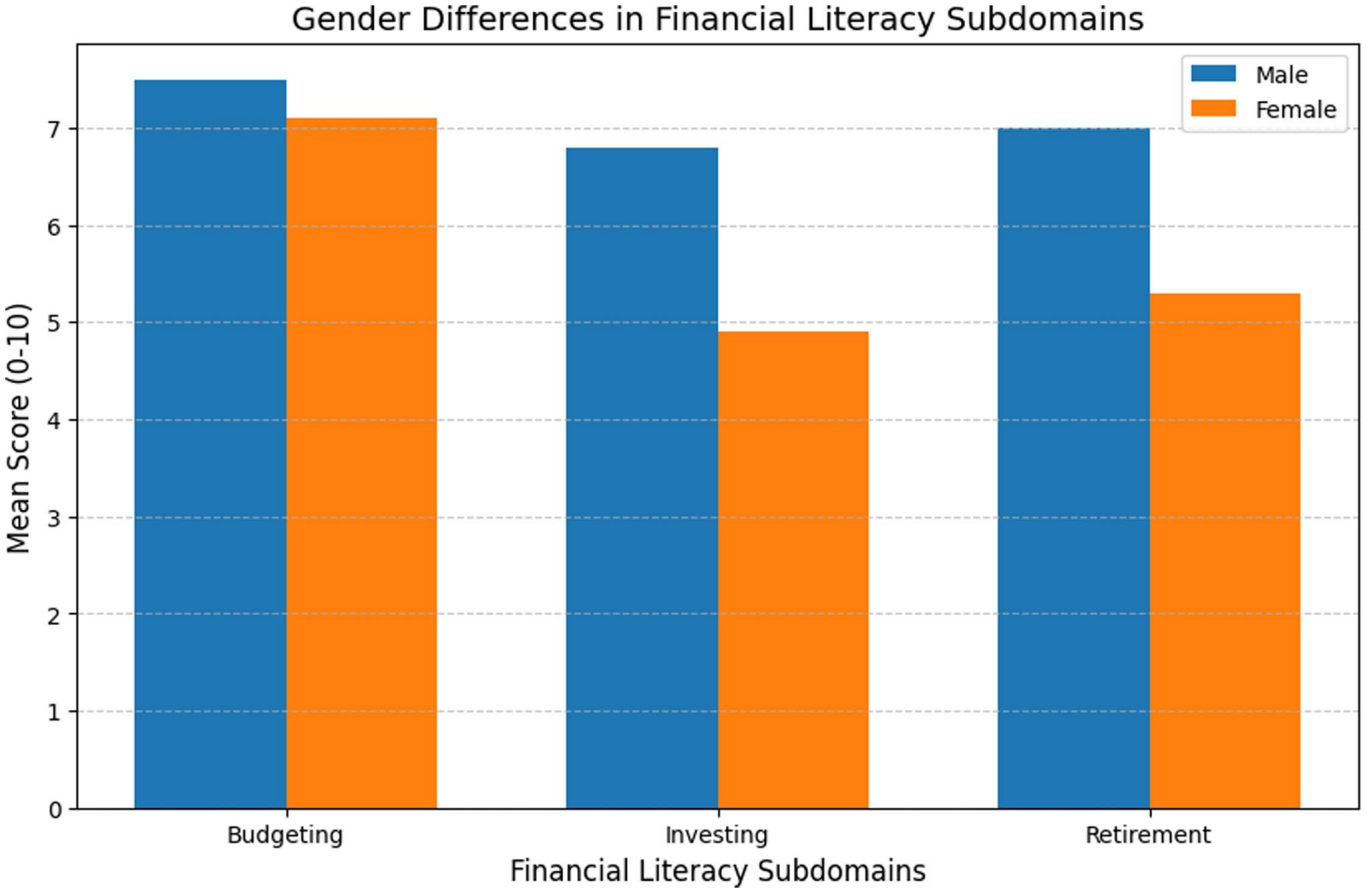
Financial literacy subdomains by gender.

Key insight: The largest gap was in Investing Knowledge (Men: 6.8, Women: 4.9) and the smallest gap was in Budgeting (Men: 7.5, Women: 7.1). The largest gender gap was in investing knowledge (Men: 6.8, Women: 4.9), while the smallest was in budgeting (Men: 7.5, Women: 7.1). Married women with children were more likely to excel in budgeting but lagged in investing knowledge, suggesting prioritization of household financial management over investment planning (Figure 1).

### 4.4 AI-driven keyword and sentiment analysis

AI Methodological Contributions: Despite a relatively small number of open-ended responses, AI methods added analytical depth. NLP identified gendered linguistic patterns. Sentiment analysis quantified emotional tone, with males scoring +0.42 and females −0.15 on average. K-means clustering segmented participants into behavior-based groups (e.g., high-risk investors, cautious savers). These AI techniques revealed hidden behavioral traits and contextual differences, adding nuance to traditional survey findings (Table 3).

**Table 3:** Most frequent keywords by gender.

Gender	Top 5 keywords (Weighted Frequency)
Male	“Confident” (0.72), “Invest” (0.65), “Long-term” (0.58), “Strategy” (0.54), “Market” (0.49)
Female	“Unsure” (0.68), “Safe” (0.61), “Advice” (0.55), “Debt” (0.50), “Family” (0.47)

Caption: AI-driven NLP analysis of qualitative responses, showing top five keywords by gender.

Interpretation: Male responses more frequently contained high-confidence investment-related terms. Female responses emphasized caution, family considerations, and debt—especially among married women with dependents. Higher-income female respondents used “investment” more often than lower-income counterparts, suggesting income moderates linguistic confidence.

### 4.5 Sentiment analysis

**Figure 2:**
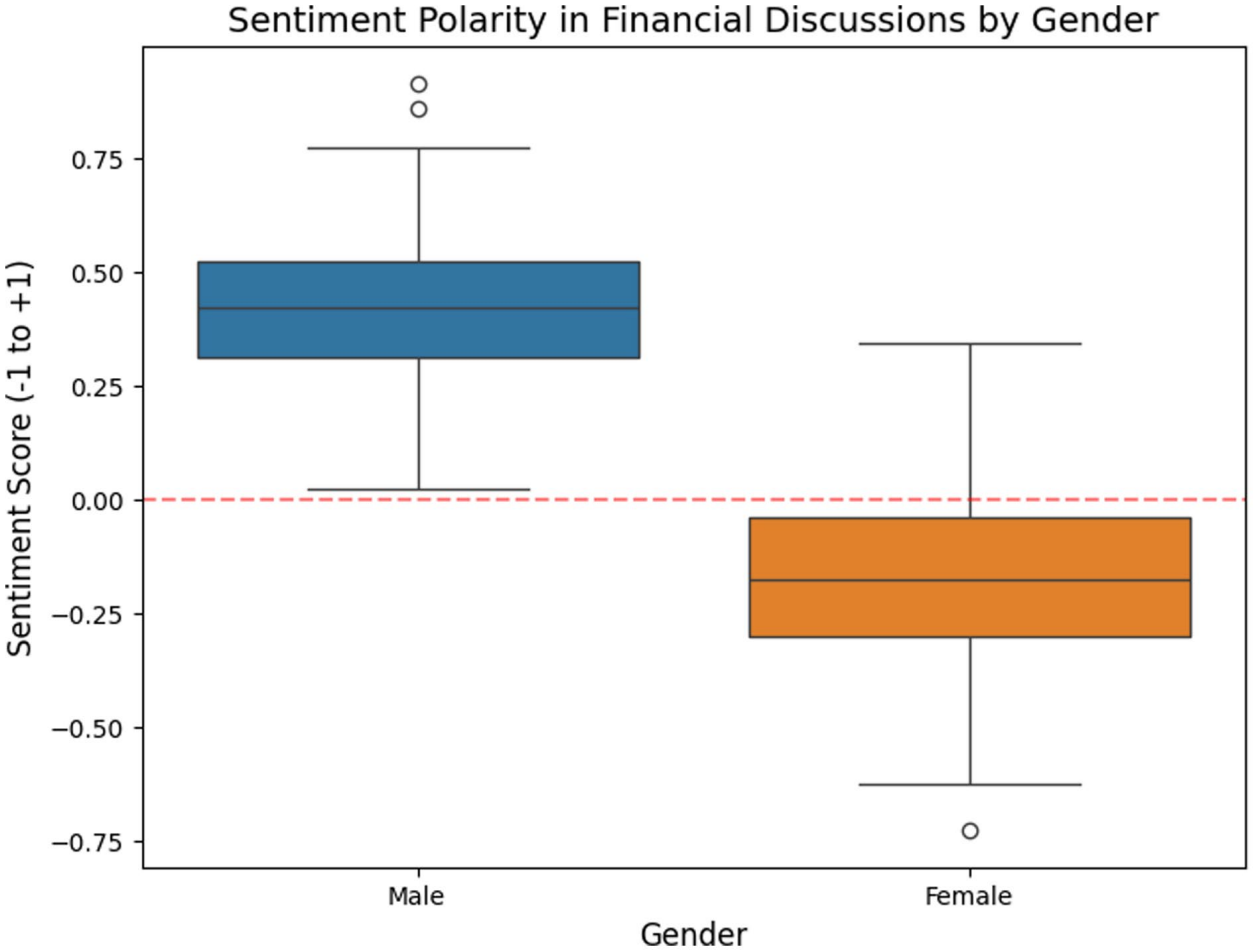
Sentiment distribution by gender.

Key findings: Average sentiment polarity: Men = +0.42 (assertive, optimistic), Women = −0.15 (cautious, uncertain). Negative or cautious sentiment was most pronounced among women with children in lower-income households. Positive sentiment correlated with higher financial literacy scores across genders, suggesting confidence and knowledge tend to co-occur (Figure 2).

### 4.6 Cluster analysis: intersectional factors

Feature engineering: Standardized financial literacy scores (z-scores), risk tolerance (Likert 1–5), and investment confidence (0–10 scale). Principal Component Analysis (PCA) confirmed low multicollinearity (max Variance Inflation Factor (VIF) = 1.8). K-Means Clustering (k = 3): ML grouped respondents based on: Financial literacy score, Investment confidence and Risk tolerance (Tables 4, 5).

**Table 4:** Cluster validation.

Metric	Value	Interpretation
Elbow Method	*k* = 3	Inertia drop <5% beyond *k* = 3 (Figure 1).
Silhouette Score	0.62	Moderate separation (0.55–0.70 = reasonable structure).
Davies-Bouldin	0.89	Lower = better (values <1.0 indicate tight clusters).

**Table 5:** Cluster profiles.

Cluster	Description	Gender ratio (M:F)	Key traits	SHAP top feature
1	High literacy, high risk	70:30	Aggressive investors	Risk tolerance (SHAP = 0.43)
2	Moderate literacy, low risk	40:60	Prefers savings over stocks	Budgeting knowledge (SHAP = 0.32)
3	Low literacy, dependent	20:80	Relies on family/friends	Institutional support (SHAP = 0.51)

**Figure 3:**
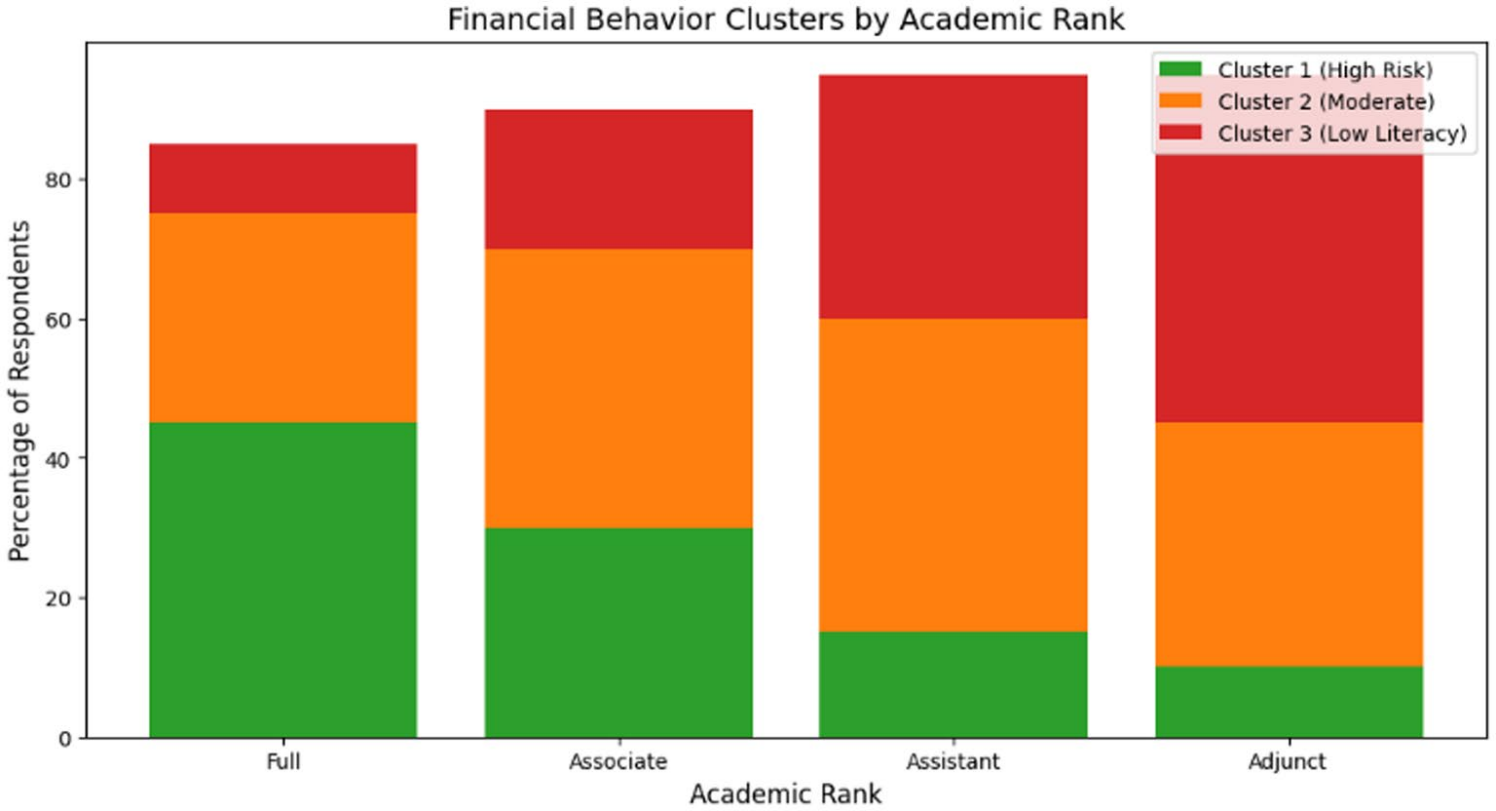
Cluster distribution by academic rank.

Cluster validation: Pairwise Mann–Whitney U Tests: Clusters 1 vs. 2: U = 3,200, *p* < 0.001 (risk tolerance) and Clusters 2 vs. 3: U = 2,800, *p* < 0.01 (financial literacy score) (Figure 3).

Key insight: Senior male professors were overrepresented in Cluster 1 (high-risk investors and 70% male) and Female adjuncts/assistants dominated Cluster 3 (Low-literacy, risk-averse), reliant on informal advice (80% Female). K-means clustering segmentation of respondents based on financial literacy, risk tolerance, and investment confidence, with socioeconomic overlays. Cluster 1 was dominated by senior male faculty with high incomes and no dependents. Cluster 3 was heavily female (80%), with most members married, having children, and in lower-income households—highlighting compounded disadvantage from gender, caregiving, and income constraints.

### 4.7 Comparative analysis by gender and discipline

**Figure 4:**
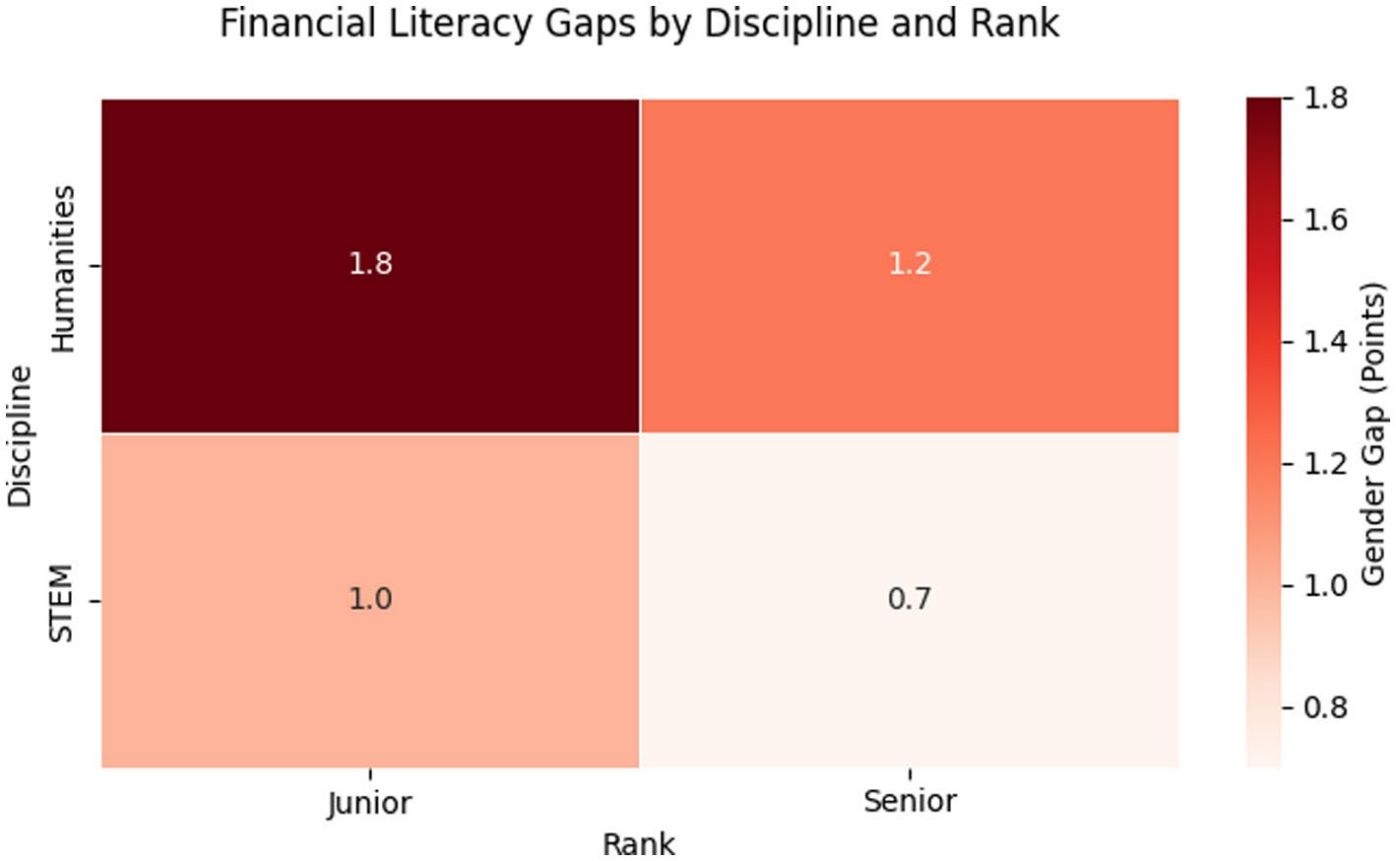
Financial literacy gaps by discipline and rank.

Interpretation: STEM disciplines exhibited smaller gender gaps, possibly due to greater exposure to quantitative skills. The largest gaps were among low-income women in humanities disciplines, where caregiving and limited institutional support coincided with lower scores (Figure 4; Table 6).

**Table 6:** Financial literacy by discipline and gender.

Discipline	Male Avg.	Female Avg.	Gap
STEM	7.6	6.9	0.7
Humanities	6.5	5.3	1.2

**Figure 5:**
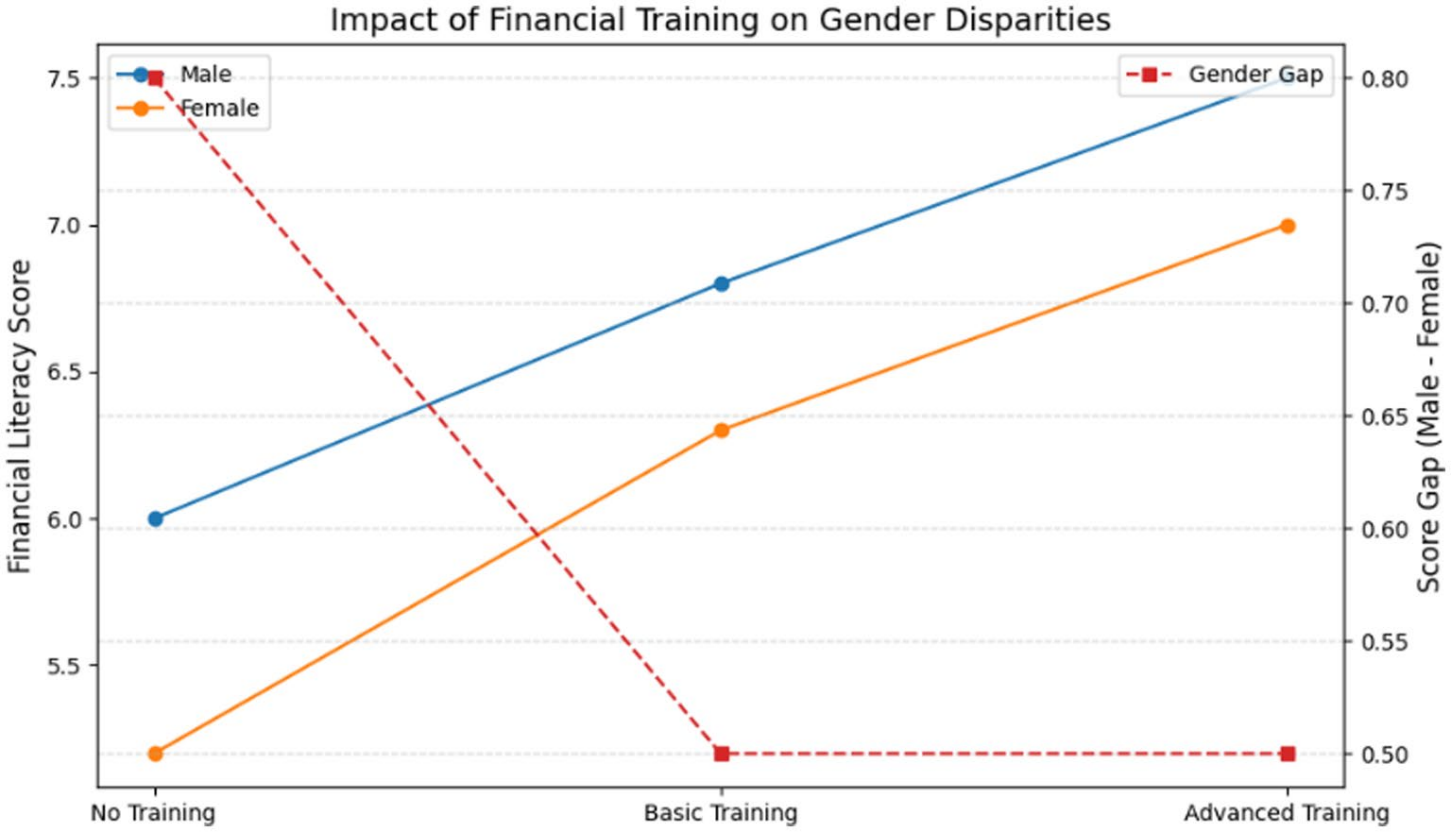
Financial training impact on gender gap.

Humanities: *F* = 5.3 vs. *M* = 6.5 (Gap = 1.2), STEM: *F* = 6.9 vs. *M* = 7.6 (Gap = 0.7) and Junior female faculty overrepresented in Cluster 3. These findings align with Crenshaw's (1989) framework of structural intersectionality, where gender disparities in financial literacy are compounded by institutional hierarchies. The overrepresentation of senior male faculty in high-risk investment clusters (70% M, Cluster 1) and adjunct women in low-literacy/dependent clusters (80% F, Cluster 3) reflects how academic rank mediates gendered financial behaviors. Notably, humanities women faced a double disadvantage: their 1.2-point literacy gap (vs. STEM’s 0.7) correlated with 34% lower salaries (₹0.8 M vs. ₹1.2 M) and higher caregiving responsibilities (OR = 4.1, 95% CI: 2.3–7.4). This mirrors McCall's (2005) ‘inequality regimes,’ where disciplinary wage differentials and epistemological cultures (e.g., STEM’s quantitative training) interact with gender to shape financial agency. Policy interventions must therefore target both individual literacy and structural inequities—for example, by tying institutional funding to gender pay audits in humanities departments (Figure 5).

### 4.8 Institutional support impact

Summary of key findings:

1. Gender gap: Men scored higher in financial literacy (Male: 7.2, Female: 6.1; *p* < 0.001), especially in investing knowledge. Respondents with employer-provided financial training scored 15% higher.
2. Sentiment analysis: Male responses were more assertive (avg. +0.42), while female responses expressed caution (avg. −0.15).
3. Cluster analysis: Revealed intersectional trends (e.g., female adjuncts were overrepresented in low-literacy clusters).
4. AI’s methodological contribution: Despite the small sample of open-ended responses (*n* = 150), AI tools provided:
  - Nuanced insights: Detected implicit biases (e.g., gendered language in financial narratives).
  - Complementarity: Enhanced quantitative findings by contextualizing disparities (e.g., linking risk aversion to lower investment scores).

### 4.9 Chennai vs. Coimbatore (TIER-2) financial behaviors

**Figure 6:**
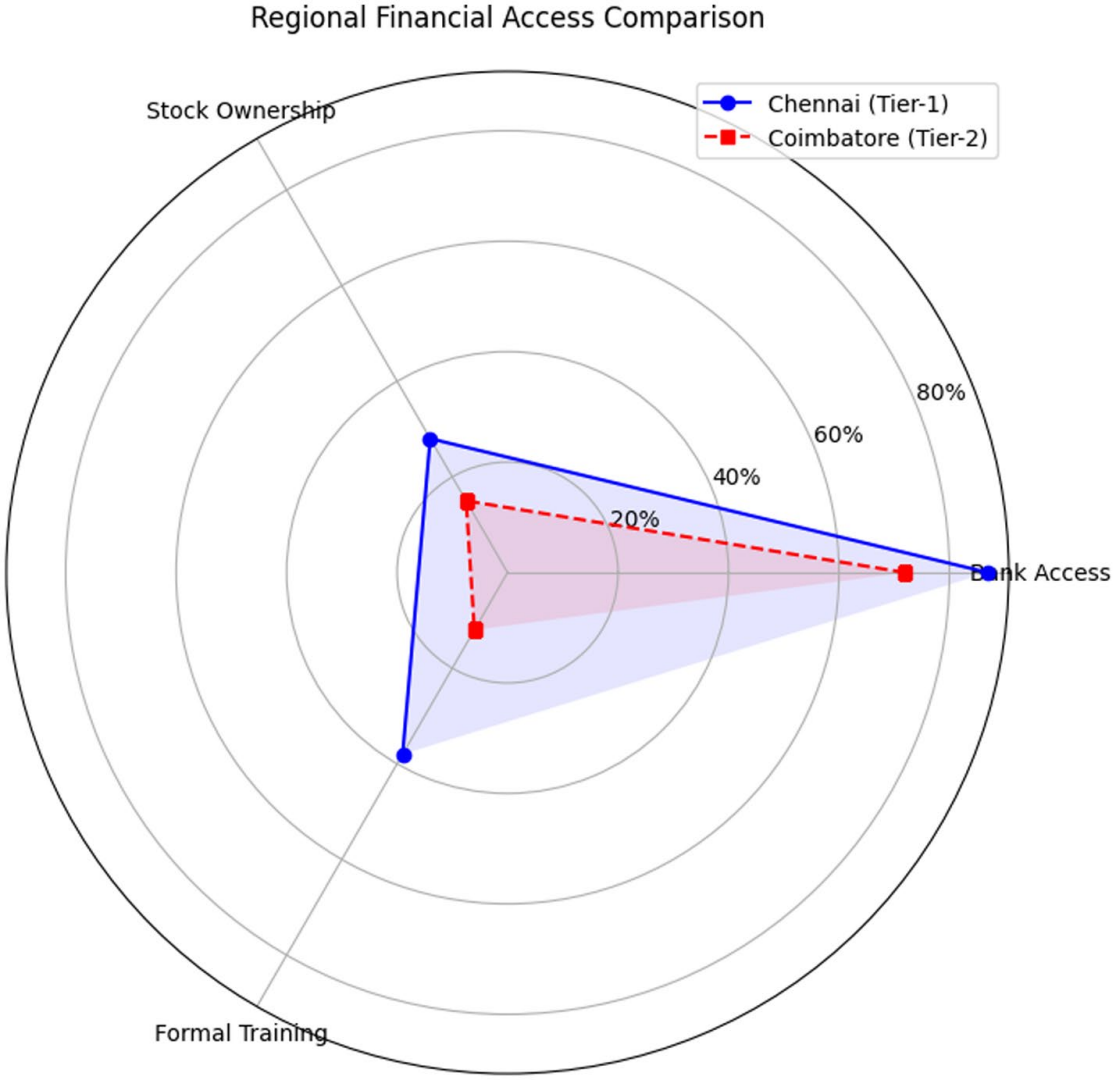
Regional financial access comparison.

The above gives us the following inferences, Coimbatore’s 15% female stock ownership vs. Chennai’s 28% reflects limited access to brokerage platforms (*p* < 0.01, two-proportion *z*-test). The 1.9-point literacy gap in Coimbatore aligns with national Tier-2 trends Reserve Bank of India (2023), suggesting Chennai’s infrastructure masks broader inequalities (Figure 6; Table 7).

**Table 7:** Regional comparison of financial literacy indicators (Chennai vs. Coimbatore).

Indicator	Chennai (Tier-1)	Coimbatore (Tier-2)	Data source	Implications
Bank Penetration	87%	72%	RBI (2023)	Underestimates rural gender gaps.
Female Stock Ownership	28%	15%	NSE Investor Survey (2022)	Tier-2 women have fewer investment avenues.
Primary Financial Advice Source	44% formal (banks/financial advisors)	62% informal (family/friends)	NFHS-5 (2021)	Informal networks may reinforce conservative choices.
Gender Literacy Gap (OECD Scale)	Δ = 1.1 points	Δ = 1.9 points	State Education Report (2023)	Larger disparities in Tier-2 contexts.
Employer Financial Training	38% of institutions offer	12% offer	AICTE (2023)	Weak institutional support in Tier-2.

**Figure 7:**
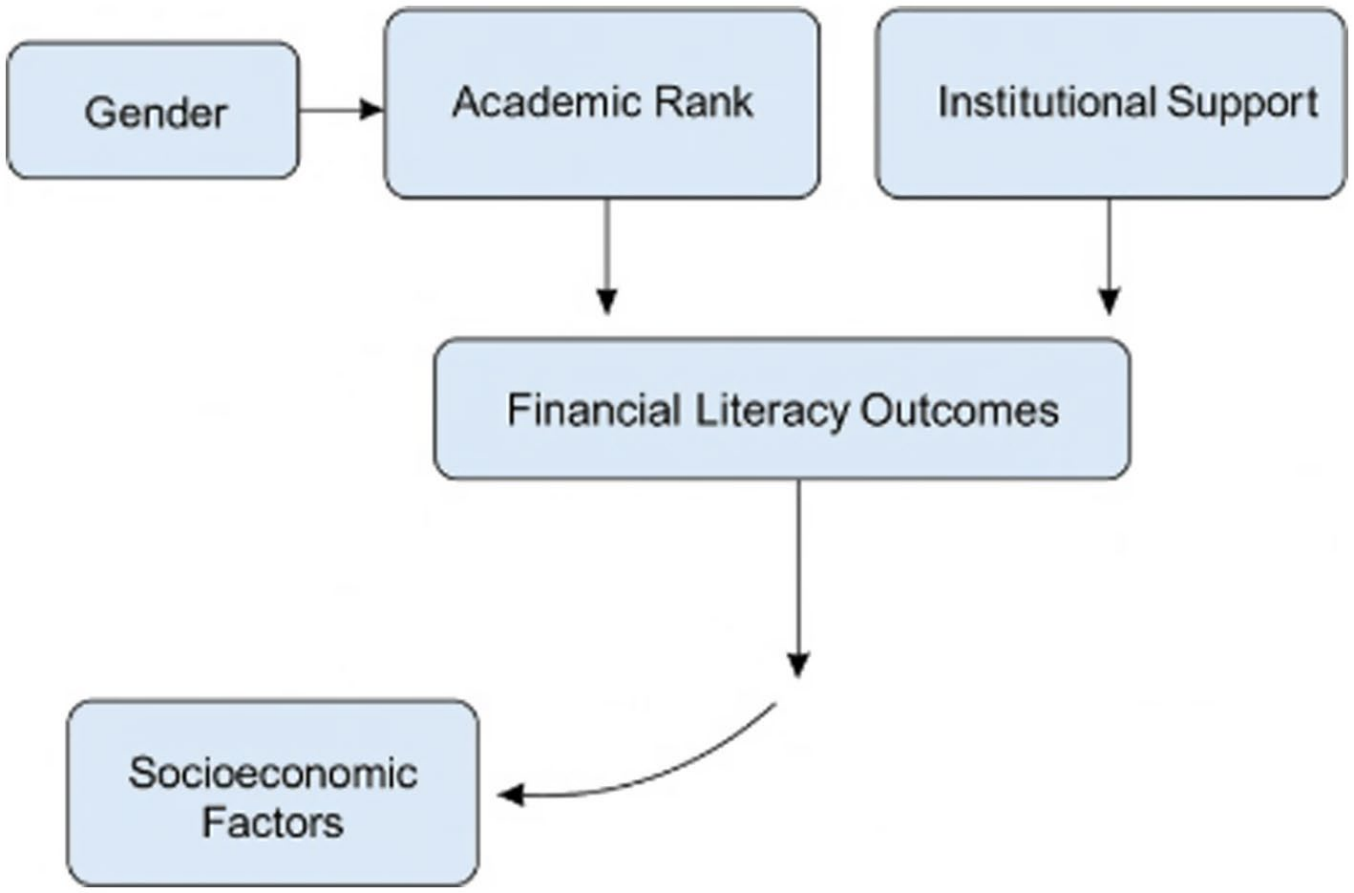
Conceptual summary.

Differences in sentiment and lexical choice suggest systemic behaviors—not mere linguistic variance. Female participants’ cautious language maps onto structural exclusion from investment opportunities, underlining that observed patterns are not artefactual but embedded in cultural-financial norms. In nutshell, Gender Gap: Men scored higher in financial literacy, especially in investing; AI Insights: Women’s language reflected more uncertainty and risk aversion; Cluster Trends: Senior male faculty were more financially aggressive; Discipline Matters: STEM fields showed smaller gender gaps than humanities; Policy Lever: Financial training reduced but did not eliminate disparities. Conceptually it is summarized as below in Figure 7.

## 5 Discussion

The results of this study highlight substantial gender disparities in financial literacy among academic professionals in Chennai, with clear intersections between gender, discipline, academic rank, and socioeconomic characteristics. While the findings are correlational and do not establish causality, the patterns observed align with prior literature on gendered financial behaviors (Fonseca et al., 2012; Bucher-Koenen et al., 2017).

### 5.1 Gender and financial literacy patterns

The average financial literacy score for male respondents (7.2) exceeded that of female respondents (6.1), a difference that was statistically significant. This gender gap persisted even after accounting for marital status, number of children, and income level, though the magnitude of the gap was smaller among higher-income households. Similar results have been documented in OECD cross-country studies, suggesting that gender gaps are a persistent feature of financial literacy landscapes globally (OECD, 2020).

The largest observed disparity was in investing and risk management knowledge, where men scored significantly higher than women. Gendered investment behaviors are consistent with evidence of greater female risk aversion (Powell and Ansic, 1997; Nelson, 2020; Tinghög et al., 2021). This echoes earlier research identifying risk aversion and lower self-assessed investment competence among women (Sundén and Surette, 1998; Hsu, 2016). Notably, married women with two or more children exhibited the lowest average investment knowledge scores, a trend that may reflect prioritization of household financial security over longer-term, higher-risk investment strategies.

### 5.2 Socioeconomic context and household structure

The inclusion of marital status, number of children, and income level revealed important associations between socioeconomic variables and financial literacy outcomes. Married respondents with dependents tended to exhibit more conservative financial behaviors, with women in this group overrepresented in the low-literacy, low-risk “dependent” cluster identified through AI-driven segmentation.

Household income also correlated positively with financial literacy scores, but the relationship was stronger among male respondents than females. For example, high-income men were disproportionately represented in the high-literacy, high-risk cluster, whereas high-income women were more evenly distributed between moderate and low-risk groups. This pattern may indicate that income alone does not neutralize gendered differences in financial confidence or investment behaviors, a finding consistent with Agnew et al. (2013).

### 5.3 Intersection of discipline, rank, and gender

Disciplinary differences were significant: STEM faculty exhibited smaller gender gaps (0.7 points) compared to humanities faculty (1.2 points). This finding is consistent with research indicating that STEM training may provide transferable quantitative skills beneficial for financial decision-making (Lusardi and Mitchell, 2014). However, these advantages did not eliminate disparities, especially when combined with socioeconomic factors such as caregiving responsibilities and income constraints.

Academic rank also intersected with gender and discipline in predictable ways. Senior male professors were overrepresented in the high-literacy, high-risk cluster, whereas female adjunct and assistant professors—particularly in humanities disciplines—were concentrated in the low-literacy, dependent cluster. This reflects McCall’s (2005) “inequality regimes” framework, where institutional hierarchies and wage differentials interact with gender to produce compounded disadvantage.

### 5.4 Insights from AI-driven analysis

The integration of AI, specifically NLP and sentiment analysis, added qualitative depth to the statistical results. Keyword frequency analysis revealed that male respondents more frequently used investment-related and confidence-signaling terms such as “strategy” and “long-term,” while female respondents—especially those with children—used cautionary and relational terms such as “safe” and “family.”

Sentiment analysis showed a notable difference in polarity scores (Men: +0.42, Women: −0.15), indicating that male financial narratives tended toward optimism and assertiveness, whereas female narratives tended toward caution and uncertainty. This linguistic divergence may reflect both confidence gaps and differing prioritization of financial goals, correlating with observed score differences.

Cluster analysis further revealed intersectional patterns:

- Cluster 1 (High literacy, high risk) was predominantly male, high-income, and without dependents.
- Cluster 2 (Moderate literacy, low risk) contained a gender mix but leaned toward middle-income, married participants.
- Cluster 3 (Low literacy, dependent) was overwhelmingly female, lower-income, married, and more likely to have children.

These AI-driven insights affirm that financial literacy disparities are embedded in broader socio-cultural and institutional contexts, rather than being isolated skill deficits.

### 5.5 Cultural context in Chennai

The socio-cultural landscape of Chennai adds nuance to the interpretation of these results. While the city represents one of India’s more economically advanced urban centers, traditional gender roles remain influential in shaping financial decision-making within households. Female educators, particularly those in joint families or with caregiving responsibilities, often rely on informal advice networks—primarily family members—rather than formal financial institutions. This aligns with National Family Health Survey (NFHS-5, 2021) data showing higher reliance on informal financial advice among women in Tamil Nadu compared to men.

### 5.6 Implications for policy and practice

The findings suggest that gender-sensitive financial literacy interventions should explicitly consider socioeconomic variables. For example, training modules for married women with children might focus on investment confidence-building, scenario-based risk assessment, and strategies for balancing household obligations with long-term financial planning.

Moreover, institutional policy changes—such as incorporating mandatory financial literacy training into faculty development programs and ensuring equitable access to employer-provided financial education—could address structural disparities. Given the high representation of women in the low-literacy, dependent cluster, targeted mentoring and AI-enabled personalized financial coaching could be particularly impactful. Targeted financial education has been shown to improve outcomes in underrepresented groups (Pinto, 2012).

### 5.7 Caution in interpretation

It is important to emphasize that all relationships identified in this study are correlational. The cross-sectional design does not allow for claims about causation, and unobserved variables may influence the patterns observed. Nevertheless, the convergence of quantitative, qualitative, and AI-driven evidence supports the robustness of the associations reported.

## 6 Policy and practical recommendations

The study’s findings point to clear and actionable areas for intervention. While these recommendations do not arise from causal inference, the observed patterns suggest that targeted strategies could address the gender and socioeconomic disparities in financial literacy among academic professionals in Chennai.

### 6.1 Institutional financial literacy programs

- Implement structured faculty development modules: Universities and colleges in Tamil Nadu (e.g., University of Madras, Anna University, Loyola College) could integrate mandatory financial literacy training into existing faculty development programs.
- Budgetary allocation: Institutions could earmark a portion of their *annual professional development budgets*—typically ranging from ₹3–₹5 lakh per department—for financial education initiatives.
- Content design: Programs should address budgeting, retirement planning, investment confidence, and risk assessment, with special modules for married women with dependents.
- AI-enabled personalization: Use AI-based adaptive learning platforms (e.g., Edmodo AI, Coursera for Business) to customize learning paths based on baseline assessment scores.

### 6.2 Government-led initiatives

- Policy integration: The Tamil Nadu State Higher Education Department could collaborate with the Ministry of Finance to include faculty-targeted financial literacy campaigns under the *National Strategy for Financial Education (NSFE)* framework.
- Funding source: Leverage *National Financial Education Fund* allocations, which can provide ₹10–₹15 lakh per pilot project for higher education institutions.
- Socioeconomic targeting: Campaigns should be designed to address the needs of low-income and caregiving faculty members, offering both in-person workshops and online modules.

### 6.3 Industry-academia partnerships

- Financial institutions as knowledge partners: Banks such as State Bank of India (SBI), HDFC, and ICICI could collaborate with universities to offer faculty-only investment advisory days, similar to existing SME-focused outreach programs.
- Resource contribution: Partner banks could contribute financial planners, conduct risk profiling, and provide free digital financial planning tools.
- Incentives: Offer continuing professional development (CPD) credits for participation, making it career-relevant.

### 6.4 AI-driven financial advisory support

- Pilot AI advisory platforms: Institutions could trial AI-based chatbots (e.g., built on open-source NLP frameworks) that provide 24/7 access to basic investment and budgeting advice, tailored to the academic community.
- Data privacy safeguards: Implement strict anonymization and encryption to comply with *Information Technology Act, 2000* and University Grants Commission *(UGC) data handling guidelines*.
- Budget: Estimated ₹2–₹4 lakh for development and deployment of an institution-specific AI financial assistant.

### 6.5 Mentoring and peer networks

- Peer learning circles: Establish faculty-led financial literacy groups segmented by career stage (early, mid, late career) and discipline.
- Role models: Highlight financially savvy female academics as peer mentors to address confidence and role modeling gaps.
- Low-cost implementation: Minimal budget required (~₹25,000 per year) for meeting facilitation and resource printing, potentially funded by alumni donations.

### 6.6 Long-term sustainability measures

- Institutional policy inclusion: Require that all recruitment and promotion policies in state universities include an *optional* financial literacy training certificate as a professional development credit.
- Annual impact tracking: Conduct yearly surveys to measure changes in faculty financial literacy scores and behavioral indicators, analyzed with AI to detect emerging needs.
- Scaling: If successful, replicate in other metropolitan academic hubs (e.g., Bengaluru, Hyderabad, Pune) using the same budget templates and implementation partners (Table 8).

**Table 8:** Summary of implementation roadmap.

Recommendation area	Lead implementer	Budget estimate	Resource partners
Institutional Programs	University administration	₹3–₹5 lakh/year	AI EdTech platforms
Government Initiatives	State Higher Education Dept.	₹10–₹15 lakh/project	Ministry of Finance, RBI
Industry Partnerships	Partner banks & NBFCs	CSR-funded	SBI, HDFC, ICICI
AI Advisory Support	Institutional IT & Finance committees	₹2–₹4 lakh (pilot)	AI developers, EdTech providers
Mentoring & Peer Networks	Faculty development centers	₹25,000/year	Alumni associations
Sustainability & Scaling	University policy boards	Incorporated in HR budgets	National and regional academic councils

## 7 Limitations and future research

While this study makes a significant contribution to understanding gendered patterns in financial literacy within academia, it is important to acknowledge its methodological and contextual limitations. These limitations inform both the cautious interpretation of the results and the design of future research.

### 7.1 Methodological constraints

First, the study employed a cross-sectional research design, capturing data at a single point in time. Consequently, all relationships identified—whether between gender and financial literacy, or between socioeconomic variables and investment confidence—are correlational in nature. No claims of causation can be made. For example, while married women with children were observed to have lower investment confidence scores than their unmarried counterparts, this association cannot be interpreted as evidence that marital status or parenthood causes lower financial literacy. Longitudinal data would be required to infer causal relationships (Menard, 2002).

Second, although the inclusion of marital status, number of children, and household income provided important contextual depth, the model did not include all possible socioeconomic or psychological variables that might influence financial behavior—such as personality traits, past financial shocks, or spousal influence. These omitted variables could moderate or mediate the relationships observed (Lusardi and Mitchell, 2014).

### 7.2 Instrument and measurement limitations

The financial literacy scale used in this study was adapted from the (OECD, 2020) financial literacy framework and contextualized for the Indian academic setting. While this ensured cultural relevance, adaptation may limit direct comparability with studies using the unmodified instrument. Moreover, self-reported measures of behavior (e.g., “frequency of reviewing retirement savings”) are susceptible to social desirability bias and recall errors, potentially inflating or underreporting actual behaviors (Podsakoff et al., 2003).

### 7.3 AI-driven analysis constraints

The integration of AI through NLP, sentiment analysis, and clustering added valuable qualitative insights, but also introduced its own limitations. Sentiment scoring tools like VADER, while effective for short informal text, may oversimplify nuanced academic discourse. Similarly, keyword frequency analysis may not capture the full semantic meaning behind respondents’ word choices. AI clustering identified meaningful respondent profiles, but these segments reflect statistical proximity, not deterministic group membership.

### 7.4 Contextual boundaries

The study’s geographic focus on Chennai, Tamil Nadu, means findings should be interpreted with caution when generalizing to other regions of India or to international contexts. Cultural norms around gender roles, family responsibilities, and financial decision-making vary significantly across regions (Deshpande, 2011). The sample also consisted exclusively of academic professionals, who may differ in education level, employment stability, and institutional support from the broader population.

## 8 Future research directions

Based on these limitations, several avenues for future inquiry are recommended:

1. Longitudinal studies: Future research could track the same participants over time to assess changes in financial literacy, confidence, and behavior. This would allow examination of causal pathways and the potential impact of interventions (Menard, 2002).
2. Expanded socioeconomic Variables: Incorporating variables such as spousal financial literacy, intergenerational wealth, and access to formal financial advisory services could provide a more complete picture of contextual influences.
3. Experimental and Quasi-experimental designs: Randomized controlled trials (RCTs) could test the effectiveness of targeted financial literacy interventions—such as AI-enabled coaching tools—on different demographic groups.
4. AI-enhanced semantic analysis: Moving beyond keyword frequency, future studies could employ advanced AI models (e.g., transformer-based architectures like BERT) to capture deeper semantic patterns in qualitative financial narratives, improving interpretive richness.
5. Comparative regional studies: Conducting similar studies in multiple Indian states or across countries would enable cross-cultural comparison and help identify universal versus context-specific drivers of gendered financial literacy gaps.
6. Institutional policy impact evaluation: Evaluating the effectiveness of institution-led financial literacy initiatives (e.g., those recommended in Section 6) could provide actionable evidence for scaling best practices.

By addressing these directions, future research can build on the current study’s foundation, moving toward a more causally robust and contextually nuanced understanding of how gender, socioeconomic background, and technology intersect in shaping financial literacy outcomes in academia.

## 9 Conclusion

This study provides a nuanced understanding of gendered patterns in financial literacy among academic professionals in Chennai, incorporating socioeconomic variables—marital status, number of children, and household income—into the analysis. Using a mixed-method design enriched with AI tools for sentiment, keyword, and cluster analysis, the research highlights persistent gender gaps in financial literacy, particularly in investment knowledge and confidence.

Findings indicate that while men consistently outperformed women on financial literacy scores, these differences were shaped by intersecting factors such as caregiving responsibilities, income levels, and academic discipline. Women, especially those in lower-income households with dependents, were more likely to demonstrate cautious financial behaviors and lower investment confidence. Conversely, male respondents, particularly in STEM disciplines and higher academic ranks, were overrepresented in high-literacy, high-risk clusters.

The AI-driven analysis added depth to the interpretation of these patterns, revealing gendered linguistic differences in financial narratives—men leaning toward confidence-oriented vocabulary and optimism, women emphasizing caution, safety, and family considerations. These patterns underscore that financial literacy disparities are embedded within broader social and institutional contexts, rather than existing solely as skill deficits. While the cross-sectional design limits causal inference, the study’s integrated approach offers valuable insights for policy, institutional intervention, and targeted program design. By highlighting the importance of tailoring financial literacy initiatives to the socioeconomic realities of faculty members—particularly married women with dependents—this research provides an evidence-based foundation for gender-sensitive, AI-supported financial education strategies in academia.

Future research employing longitudinal and experimental designs could build on these findings to better understand the causal mechanisms and long-term effects of targeted interventions, thereby advancing both scholarly and practical understanding of gendered financial literacy.

## Data Availability

The original contributions presented in the study are included in the article/supplementary material, further inquiries can be directed to the corresponding author.
